# Berberine-based carbon dots for selective and safe cancer theranostics†

**DOI:** 10.1039/c7ra12069a

**Published:** 2018-01-03

**Authors:** Fan Zhang, Ming Zhang, Xiao Zheng, Songyuan Tao, Zhanqiang Zhang, Madi Sun, Yubin Song, Jing Zhang, Dan Shao, Kan He, Jing Li, Bai Yang, Li Chen

**Affiliations:** Department of Pharmacology, Nanomedicine Engineering Laboratory of Jilin Province, College of Basic Medical Sciences, Jilin University Changchun 130021 China stanauagate@outlook.com chenl@jlu.edu.cn; State Key Laboratory of Supramolecular Structure and Materials, College of Chemistry, Jilin University Changchun 130012 P. R. China byangchem@jlu.edu.cn; School of Nursing, Jilin University Changchun 130021 China

## Abstract

Fluorescent berberine-based carbon dots (Ber–CDs) were prepared through a facile synthesis strategy. Ber–CDs exhibited excellent optical properties for bioimaging and retained the biofunctions of berberine, and enabled selective and safe anti-tumor performance, demonstrating their promising application potential in cancer theranostics.

## Abstract 2

Fluorescent berberine-based carbon dots (Ber–CDs) were prepared through a facile synthesis strategy.

## Graphical abstract

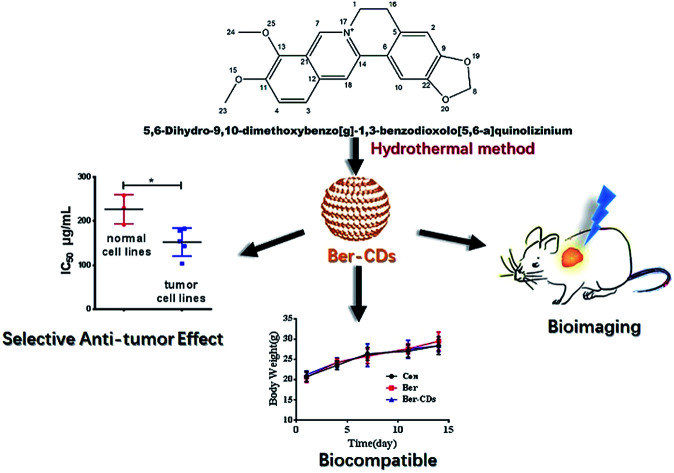

## 1. Introduction

Carbon dots (CDs) have emerged as indispensable nanomaterial owing to their unique and tunable optical properties, easy surface modification, high biocompatibility, as well as superior chemical and optical stability.^1–7^ Taking advantage over traditional organic dyes in terms of optical photostability and over semiconductor QDs concerning toxicity, CDs have been utilized as multifunctional nanocarriers for drug delivery, fluorescent imaging, and photothermal therapy in several diseases.^8–19^ Up to now, various synthetic strategies have been employed to fabricate CDs, including bottom-up carbonization from small molecules and polymers, as well as top-down cutting from different carbon sources.^1,2,20^ It is more conducive to manipulate physical characters and chemical compositions of CDs by bottom-up compared with top-down methods.^21,22^ To endow intrinsic biofunctions of CDs, biologically active precursors including small molecular drugs, proteins and nucleic acids were widely applied as carbon resources for bottom-up syntheses.^23–25^ Previous studies have showed that the combination of CDs with several anticancer agents through absorption or conjugation could be applied for cancer theranostics.^10,26–29^ In this regard, CDs could only act as diagnosis agents and limited attention has been paid to their own therapeutic properties. Hence, developing efficient and safe CDs for intrinsic cancer theranostics is highly desired.

**Scheme 1:**
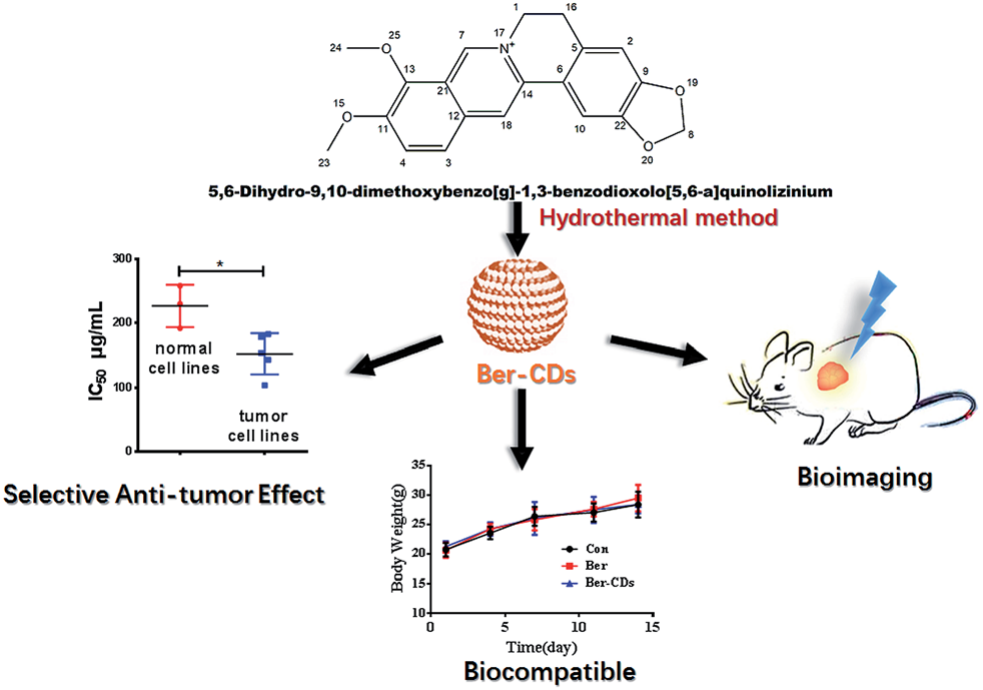
Schematic illustration for the preparation of Ber–CDs and their cancer theranostic application.

Berberine (Ber) is an isoquinoline alkaloid from the berberis species, and it exhibits extensive biomedical benefits, including anti-cancer, anti-bacterial and anti-inflammation effects.^30–32^ Besides its promising performance in cancer chemotherapy, berberine could inhibit several undesirable outcomes including drug resistance, metastasis and recurrence through reprogramming AMPK-mediated cellular energy metabolism.^33–36^ However, poor solubility and low gastrointestinal absorption have limited its clinical translation.^27,28^ To surmount these obstacles, continuous efforts have been made to improve the solubility of berberine and to increase its accumulation at targeted sites, ultimately improve the efficacy and safety of berberine administration.^32,37,38^ With these previous findings in mind, we hypothesized that berberine could be selected as precursor to fabricate CDs with the reserve of instinct pharmacological activities. In the present study, we have developed berberine-based carbon dots (Ber–CDs) through a facile strategy. To test our hypothesis, the anti-cancer ability, bioimaging capability, biocompatibility of Ber–CDs are investigated and compared with free berberine (Scheme 1).

## 2. Materials and methods

### 2.1 Reagents

Berberine (purity > 99%) was donated by the Northeast Pharmaceutical Group Co., Ltd. (Shenyang, China). 3-(4,5-Dimethylthiazol-2-yl)-2,5-diphenyltetrazolium bromide (MTT) were obtained from Sigma-Aldrich Inc. (St. Louis, MO, USA). Roswell Park Memorial Institute-1642 medium (RPMI-1640) was obtained from GIBCO (Carlsbad, CA, USA). Fetal bovine serum (FBS) was purchased from Beijing Dingguo Biological Technology Co., Ltd. (Beijing, China). Diagnostic kits for aspartate aminotransferase (AST), alanine aminotransferase (ALT), blood urea nitrogen (BUN), creatinine (CRE), triglyceride (TG), albumin (ALB), alkaline phosphatase (ALP), and total cholesterol (TC) were purchased from the Nanjing Jiancheng Bioengineering Institute (Nanjing, People's Republic of China). All the reagents were used without further purification. Deionized water was used in all experiments.

### 2.2 Synthesis of Ber–CDs

Ber–CDs were synthesized by hydrothermal methods according to our previous work.^6,24^ In general, 20 mg of berberine was mixed with 20 mL deionized water and stirred for 20 minutes to disperse. Then the turbid liquid was transferred a poly (tetrafluoroethylene) (Teflon)-lined autoclave (25 mL) and heated at 170 °C for 10 h in oven. Next the system was cooled down to room temperature and dialyzed for 72 hours in deionized water. Brown powder was obtained after freeze-drying.

### 2.3 Characterization of Ber–CDs

Transmission electron microscopy (TEM) was conducted using a Hitachi H-800 electron microscope at an acceleration voltage of 200 kV. High-resolution TEM (HRTEM) imaging was implemented by a JEM-2100F electron microscope at 200 kV. Fluorescence spectroscopy was performed using a Shimadzu RF-5301 PC spectrophotometer. UV-vis absorption spectra were obtained using a Shimadzu 3100 UV-vis spectrophotometer. Fourier transform infrared (FTIR) spectra were performed with a Nicolet AVATAR 360 FTIR instrument.

### 2.4 Cell culture

Human liver cancer HepG2 cell, human liver cancer SMMC-7721 cell, human lung cancer A549 cell, human breast cancer MCF-7, and mouse liver cancer H22 cell, as well as normal cell lines including human hepatic embryo HL-7702 cell, human umbilical vein endothelial HUVEC cell, and mouse myoblast cell C2C12 were obtained from ATCC and cultured in RPMI-1640 medium containing 10% fetal bovine serum (FBS), supplemented with 50 U mL^−1^ penicillin and 50 U mL^−1^ streptomycin, and incubated at 37 °C in 5% CO_2_ atmosphere. Cells were passaged with 0.25% trypsin/EDTA every 3 days.

### 2.5 *In vitro* toxicity studies and determination of the half maximal inhibitory concentration (IC_50_)

Cellular toxicity was determined by measuring the activity of mitochondrial enzymes in live cells to transform the soluble, yellow MTT solution to an insoluble, purple formazan product. The cells were cultured in 96-well tissue culture plates at a density of 5 × 10^3^ cells per well. After attachment, the cells were incubated with a medium containing different doses of CDs for 48 h. Subsequently after incubation, the medium from each well was removed and the cells were washed in phosphate-buffered solution (PBS). A fresh medium containing 10 μL of 5 mg mL^−1^ solution of MTT was added to each well and kept for 4 h. The medium was removed and 150 μL of DMSO was then added to dissolve the formazan crystals. The absorbance of each well was recorded at 490 nm using a multi-mode microplate reader. The untreated cells were used as controls for calculating the relative percentage cell viability [mean (%) ± SD, *n* = 6] from the following equation:Cell viability (%) = (*A*_490_ in treated sample/*A*_490_ in control sample) × 100%

The ratio of cell proliferation to control group was calculated from the MTT data. IC_50_ values were calculated from percentages of cell viability obtained from the MTT assay.

### 2.6 Cellular uptake

To explore the bioimaging potential and cellular distribution of Ber–CDs, HepG2 cells or HL-7702 cells were seeded at a density of 5 × 10^4^ in 1 mL RPMI-1640 in 24-well plate. Consequently, a fresh medium containing 25 μg mL^−1^ filtered sterilized Ber–CDs was added to the respective dishes and incubated for 3 h. After washing with PBS twice, the cells were stained with DAPI and LysoTracker Red. After incubated for 15 min, cells were imaged by using a laser scanning confocal microscopy (CLSM). For flow cytometry, Ber–CDs treated, free berberine treated and untreated cells were trypsinized, collected and resuspended in PBS for analysis. The cell suspension was then analyzed by using a flow cytometer under 488 nm excitation laser, by selecting the appropriate channel. Collected data (10 000 events per sample) were analyzed using FlowJo software.

### 2.7 Animals

All animals received care in compliance with the guidelines outlined in the Guide for the Care and Use of Laboratory Animals and the procedures were approved by the Jilin University of China Animal Care and Use Committee. Animal experiments were performed according to the guidelines of the National Institute of Health. Male NU/NU mice and ICR mice (6–8 weeks old) were purchased from the Beijing Vital River Laboratory Animal Technology Co., Ltd. The animals were maintained in specific pathogen free (SPF) animal lab.

### 2.8 *In vivo* and *ex vivo* imaging

A human liver cancer xenograft tumor model was generated through subcutaneous injection of HepG2 cells (1.5 × 10^6^, 100 μL in PBS) in the right flank of each nude mouse. For *in vivo* fluorescent imaging of HepG2 tumor-bearing mice, Ber–CDs solutions were injected directly into xenografts and visualized using an *in vivo* Imaging System at excitation and emission wavelengths of 350 and 530 nm, respectively. To reveal Ber–CDs' *ex vivo* imaging properties, HepG2 tumor-bearing mice was injected with Ber–CDs *via* tail vein (5 mg kg^−1^). After the injection, mice were sacrificed at 24 h, the tumor and major organs (heart, kidney, liver, lung and spleen) were collected and washed with PBS. For each group, three mice were used. The excised organs were visualized using the same protocol.

### 2.9 *In vivo* biodistribution of Ber–CDs

To investigate the biodistribution behaviours of Ber–CDs, blood, tumor, heart, kidney, liver, lung and spleen were collected from above-mentioned HepG2 tumor bearing mice (*n* = 6) after 24 h of injection. All weighed organs were stored and homogenized in saline and centrifuged at 3500 rpm for 10 minutes. The supernatants were collected and examined by a spectro-fluorophotometer with a 360 nm excitation wavelength, and then the photoluminescence (PL) intensity of the emission spectra at 500 nm were recorded.

### 2.10 Half-life determination of Ber–CDs

Twenty-four healthy ICR mice were fasted for 12 hours before drug administration and were given free access to water. Three mice were randomly selected at each time point. After tail vein injection of Ber–CDs, blood was taken at 0.2, 0.5, 1, 2, 4, 6, 12 and 24 h and placed in a centrifuge tube containing 10 μL of heparin and centrifuged at 4000 rpm for 10 min to draw the supernatant. The plasma samples were stored at −20 °C to be determined. The fluorescence intensity of carbon dots in plasma was measured by a fluorescence indexer and the relative fluorescence intensity *versus* time was plotted. The half-life was calculated using PKSolver software.

### 2.11 *In vivo* anti-cancer efficiency of Ber–Cds

A mouse liver cancer xenograft tumor model was generated through subcutaneous injection of H22 cells (1.5 × 10^6^, 100 μL in PBS) in the right flank of each mouse. When the tumor volume reached approximately 20–50 mm^3^, mice were randomly divided into 3 groups (*n* = 6), the treatment was started and this day was designated as day 0. Mice were treated with PBS (Con), berberine (50 mg kg^−1^ per day by i.g.), Ber–CDs (50 mg kg^−1^ per 3 days by i.v.). Tumor volume and body weight were measured twice every week to evaluate the anti-tumor activity and systematic toxicity. The estimated tumor volume (mm^3^) was calculated based on the formula length × width^2^ × 0.52.

### 2.12 Histopathology analysis and serum biochemistry assays

At day 14, mice were sacrificed, and blood was collected for serum biochemistry tests. Tumor and main organs including liver, spleen, kidney, heart, lung, and brain were excised and fixed 4% buffered paraformaldehyde overnight, and then embedded in paraffin. The paraffin-embedded tissue samples of the implanted tumor were sliced at 5 mm thickness, and stained with hematoxylin and eosin (H&E) for evaluating histopathological changes under a microscope.

### 2.13 Statistical analysis

All experiments were performed at least three times and expressed as mean ± SD. Statistical significance (*p* < 0.05) was evaluated using the Student *t*-test when only two groups were compared. If more than two groups were compared, evaluation of significance was performed using one-way ANOVA analysis of variance, followed by Bonferroni's post hoc test.

## 3. Results and discussion

### 3.1 Synthesis and characterizations of Ber–CDs

**Fig. 1:**
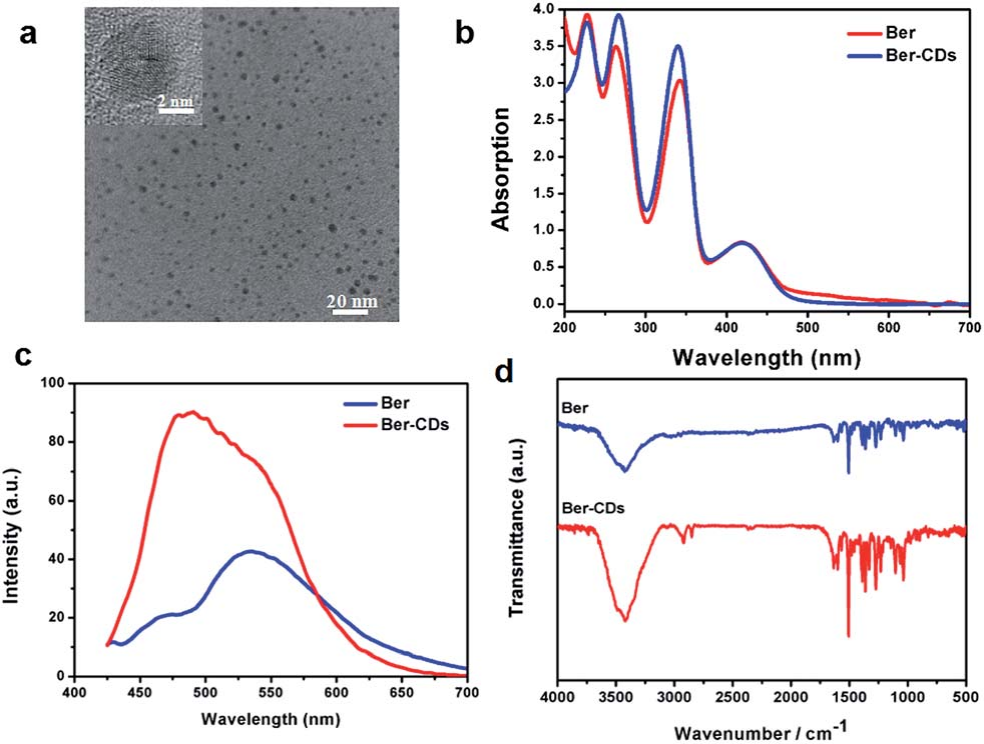
Characterization of Ber–CDs. (a) TEM and HRTEM (inset) images, (b) UV-vis absorption spectra, (c) photoluminescence spectra, and (d) FTIR spectra of Ber–CDs.

With the aid of berberine as a carbon source alone, water-soluble CDs were fabricated through a green and facile hydrothermal approach in one pot. The morphology was identified firstly through transmission electron microscopy (TEM). Ber–CDs possessed uniform dispersion without any apparent aggregation (Fig. 1a). The diameter of Ber–CDs was between 2 and 5 nm (Fig. S1, ESI†). The HRTEM image (Fig. 1a inset) also showed that Ber–CDs contained a highly carbon crystalline core with a lattice spacing of approximately 0.33 nm, which represented the (002) facet of graphite. The hydrodynamic size of Ber–CDs was 11.5 nm (Fig. S2†). As shown in Fig. 1b, the UV-vis absorption spectrum of berberine solution showed that four absorption peaks that were found at the wavelengths of 227 nm, 262 nm, 345 nm, and 420 nm. They were corresponding to n–σ transition, π–π* transition of aromatic ring, π–π* transition of aromatic ring, and n–π* transition.^38^ After hydrothermal processing, Ber–CDs possessed similar absorption spectrum to berberine. It is worth noting that a stronger absorption peak of Ber–CDs centred around 262 nm and 345 nm implied the dominance of electrostatic interactions between the aromatic rings of berberine molecules themselves. As shown in Fig. 1c, Ber–CDs exhibited broad range of emission wavelengths while the strongest fluorescence emission was centred at 490 nm under 380 nm excitation wavelengths, which showed a slight red shift when compared with berberine solution. This phenomenon might due to optical selection of different surface defect states near the Fermi level of CDs.^2^ Ber–CDs solutions could be stored for as long as 30 days with less precipitates or fluorescence loss, demonstrating its good photostability (Fig. S3, ESI†). Moreover, the Ber–CDs exhibited good fluorescent stability in acidic solution (Fig. S4†). To determine if the bioactive groups of berberine could be reserved after CDs formation, FTIR spectra were adopted to further analyze the chemical structure of Ber–CDs. As shown in Fig. 1d, Ber–CDs exhibited C=N stretching vibration of quaternary amine center at 1670 cm^−1^ and *ϕ*–C–O stretching vibration of benzyloxy at 1270 cm^−1^, which preserved the effective bioactive groups of berberine. The results of XPS analysis have shown the presence of nitrogen element as well (Fig. S5†). Collectively, these characteristics suggest that highly fluorescent Ber–CDs exhibited good optical properties, leading to promising opportunities for its potential use in biomedical applications.

### 3.2 Bioimaging of Ber–CDs

**Fig. 2:**
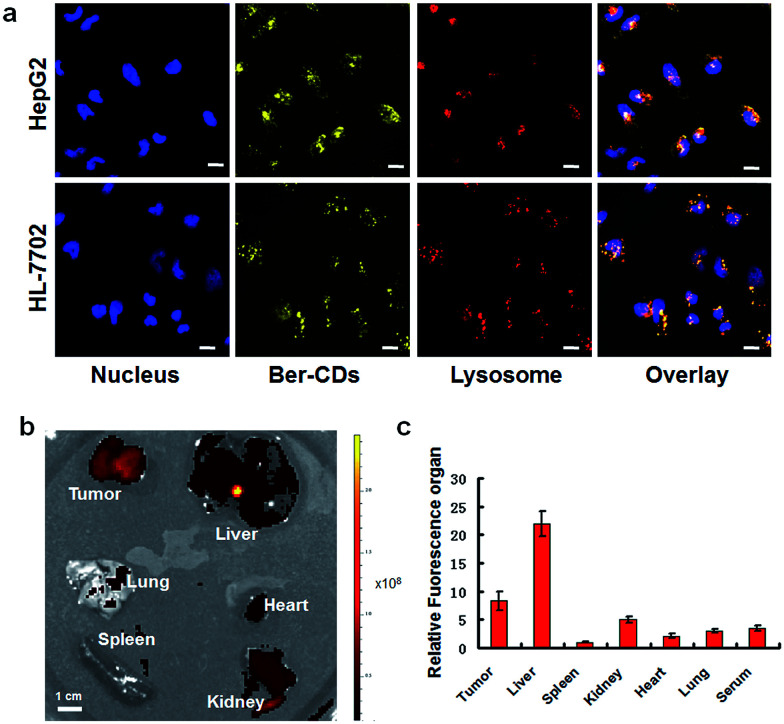
Fluorescent imaging of Ber–CDs. (a) CLSM images of HepG2 and HL-7702 cells after incubating with Ber–CDs (25 μg mL^−1^) for 3 h, scale bars represent 10 μm. (b) *Ex vivo* fluorescent imaging of Ber–CDs (5 mg kg^−1^) in different organs of HepG2 tumor-bearing mice. (c) Biodistributions of Ber–CDs based on the fluorescence intensity of organ and serum. Each bar represents the mean ± SD, *n* = 6.

The potential cell imaging application of the prepared Ber–CDs was firstly evaluated through laser scanning confocal microscopy (CLSM) on HepG2 cells and HL-7702 cells. Consistent with our previous reports,^13,39^ Ber–CDs has been internalized into cytoplasm and co-localized with endosomes or lysosomes after 3 h incubation in both HepG2 and HL-7702 cells (Fig. 2a), meanwhile the differences of fluorescent intensity between these two types of cells demonstrated that the internalization of Ber–CDs in HL-7702 cells was less than that in HepG2 cells. Importantly, quantitative fluorescence activated cell sorter (FACS) analysis demonstrated that the internalization of Ber–CDs in HepG2 cells was significantly higher than that in HL-7702 cells (Fig. S6, ESI†), which was consistent with CLSM images. To further reveal its *in vivo* imaging properties, Ber–CDs were injected directly into the tumor site using nude mice bearing HepG2 xenografts, and fluorescence can be monitored at tumor site (Fig. S7, ESI†). Valid fluorescence of Ber–CDs were observed in the tumor area, indicating its passive tumor accumulation properties.^10^ After 24 h of post injection, mice were sacrificed and liver, spleen, kidney, heart, lung, and tumor were excised for *ex vivo* fluorescence imaging (Fig. 2b). As shown in Fig. 2c, stronger fluorescence signals of Ber–CDs were observed in tumor, liver and kidney rather than the other organs. These findings further demonstrated that Ber–CDs could passively targeted the tumor site *via* the well-known enhanced permeability and retention (EPR) effect, as well as being accumulated in reticuloendothelial system (RES) organs including liver and kidney due to their smaller size.^8,10,23^ Moreover, we used the fluorescent method to investigate the circulation half-life of Ber–CDs *in vivo*. As shown in Fig. S8,† after fitting to a two-phase decay model, it was apparent that the half-life of Ber–CDs was 10.74 h.

### 3.3 The anti-tumor effects of Ber–CDs

**Fig. 3:**
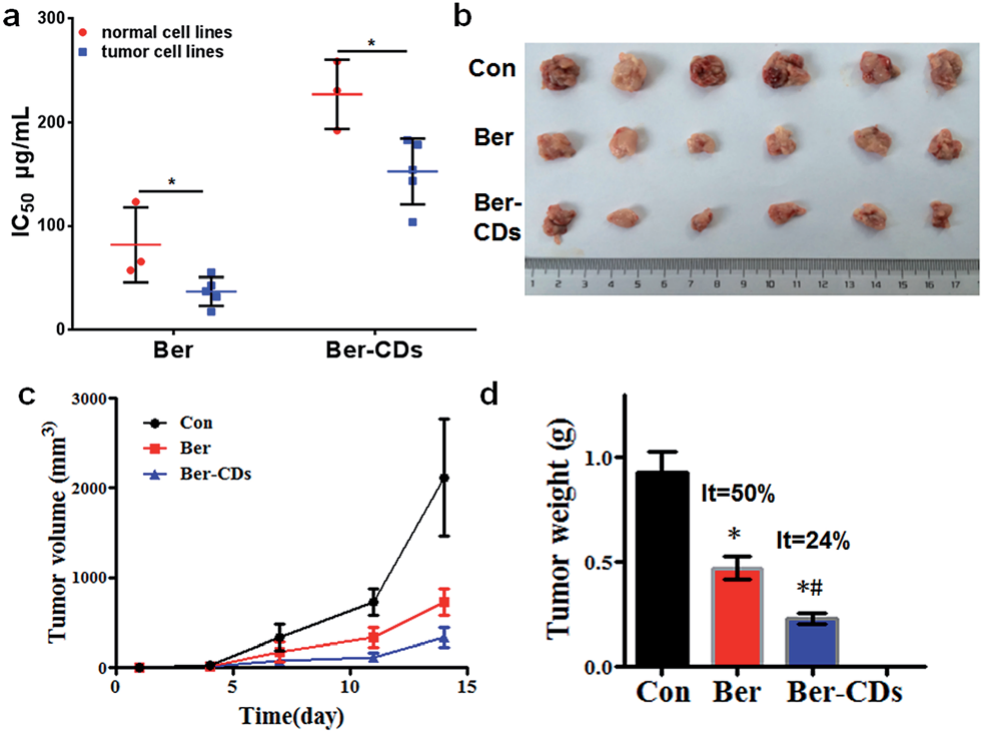
Anti-tumor effect of Ber–CDs. (a) IC_50_ values of Ber or Ber–CDs on cancer cell lines (HepG2, MCF-7, A549, SMC-7721, and H22) and normal cell lines (HL-7702, HUVEC, and C2C12) for 48 h. (b) Tumor picture, (c) tumor volume, and (d) tumor weight from mice after administration of Ber or Ber–CDs. Each bar represents the mean ± SD, *n* = 6, and *P < 0.05 *vs.* the control group, ^#^*P* < 0.05 *vs.* Ber group.

Given that Ber–CDs still retained the bioactive groups of berberine, we next investigated the *in vitro* anti-cancer effects of prepared Ber–CDs through methylthiazolyldiphenyl-tetrazolium bromide (MTT) assay. The results suggested that both Ber–CDs and free berberine could dose-dependently decreased cell viability of cancer cell lines including human liver cancer HepG2 cell, human liver cancer SMMC-7721 cell, human lung cancer A549 cell, human breast cancer MCF-7 cell, and mouse liver cancer H22 cell, as well as normal cell lines including human hepatic embryo HL-7702 cell, human umbilical vein endothelial HUVEC cell, and mouse myoblast C2C12 cell (Fig. S9–16, ESI†). The IC_50_ of Ber–CDs and free berberine on aboved cells were depicted in Fig. 3a, cancer cell lines were more susceptible to Ber–CDs rather than normal cell lines, indicating the selective anti-cancer performance of Ber–CDs. This interesting finding might be attributed to the similar anti-cancer effects of berberine.^32,40^ Although Ber–CDs showed lower anti-cancer effect than free berberine, its high water solubility and cancer cells specificity might achieve efficient and safe therapeutic performance *in vivo*. As shown in Fig. 3b and c, the *in vivo* tumor growth was significantly delayed after 14 days in the Ber–CDs treated group when compared with free berberine and control group. The smaller tumor size and more decrease of tumor weight also confirmed better anti-tumor efficacy of Ber–CDs rather than free berberine (Fig. 3b and d).

**Fig. 4:**
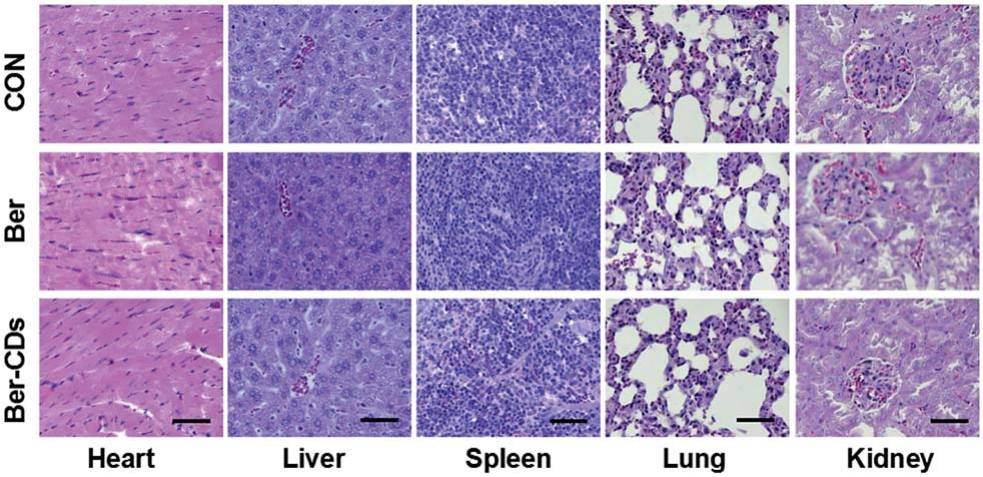
Representative H&E stained images of heart, liver, spleen, and kidney from mice in the control, Ber and Ber–CDs groups at 14 days. Scale bars represent 100 μm.

Finally, we performed a toxicological evaluation through comparing the body weight, biochemical parameters and organ histopathology of different groups at the end of treatment. No remarkable change was found in body weight in mice from all groups (Fig. S17, ESI†). Results of several serum biochemistry assays including alanine aminotransferase (ALT), aspartate aminotransferase (AST), blood urea nitrogen (BUN), creatinine (CRE), triglyceride (TG), albumin (ALB), alkaline phosphatase (ALP), and total cholesterol (TC) from blood samples demonstrated no significant differences for mice in all three groups (Fig. S18, ESI†). In addition, pathological damages were not observed in liver, spleen, kidney, heart and lung (Fig. 4). Taken together, Ber–CDs have greatly inhibited tumor growth with good biocompatibility.

## 4. Conclusions

In summary, we fabricated a kind of novel multifunctional Berberine-based CDs through a facile bottom-up strategy. Ber–CDs possessed good optical properties and retained the bioactive groups of berberine. Ber–CDs can be internalized into cancer cells and achieved targeted bioimaging *in vivo*. Importantly, Ber–CDs selectively killed cancer cells and inhibited tumor growth significantly without any obvious toxicity. This work suggests that berberine-based CDs as a selective and safe nanocarrier for cancer theranostics and show considerable potential in biomedical applications.

## Conflicts of interest

There are no conflicts to declare.

## Supplementary Material

RA-008-C7RA12069A-s001
