# Supplementary material for: Berberine-based carbon dots for selective and safe cancer theranostics
Source: RSC Adv. 2018 Jan 3;8(3):1168–73. doi: 10.1039/c7ra12069a (PMC9076993; doi:10.1039/c7ra12069a)
Supplement: RA-008-C7RA12069A-s001 [file RA-008-C7RA12069A-s001.pdf]

Electronic Supplementary Material (ESI) for RSC Advances.
This journal is © The Royal Society of Chemistry 2017

Supporting Information

Berberine-based Carbon Dots for Efficient and Safe Cancer Theranostics

Fan Zhang,‡[a] Ming Zhang,‡[a] Xiao Zheng,[a] Songyuan Tao,[b] Zhanqiang Zhang,[a] Madi Sun,[a] Yubin Song,[b] Jing Zhang,[a] Dan Shao,*[a] Kan He,[a] Jing Li,[a] Bai Yang*[b] and Li Chen*[a,c]

[a]Department of Pharmacology, Nanomedicine Engineering Laboratory of Jilin Province, College of Basic Medical Sciences, Jilin University, Changchun 130021, China. E-mail: stanauagate@outlook.com (D. S.) and chenl@jlu.edu.cn (L. C.)

[b]State Key Laboratory of Supramolecular Structure and Materials, College of Chemistry, Jilin University, Changchun, 130012, P. R. China. E-mail: byangchem@jlu.edu.cn

[c]School of Nursing, Jilin University, Changchun 130021, China

‡These authors contributed equally.

Figure Caption

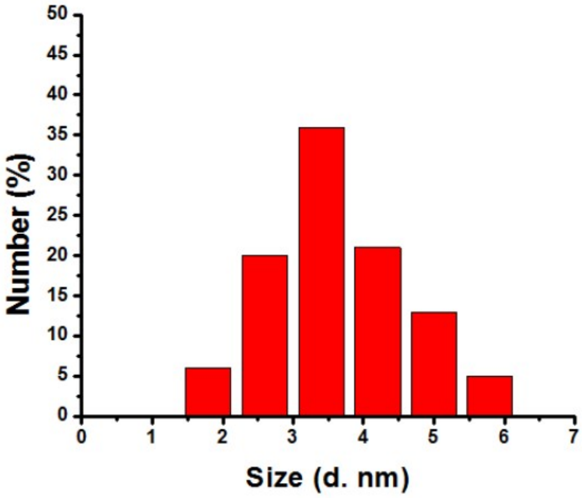

Figure S1. Size distributions of Ber-CDs.

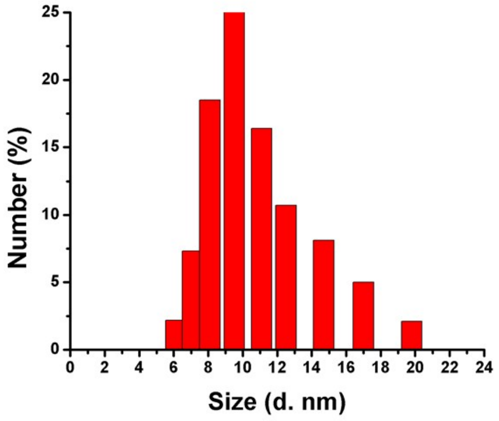

Figure S2. The hydrodynamic size of Ber-CDs.

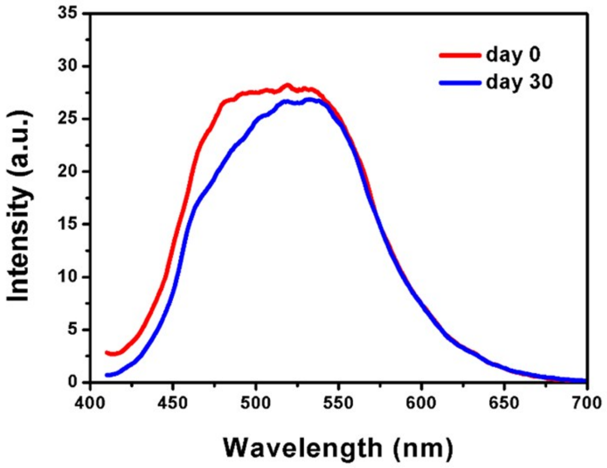

Figure S3. Photostability data for the Ber-CDs stored at room temperature for 30 days.

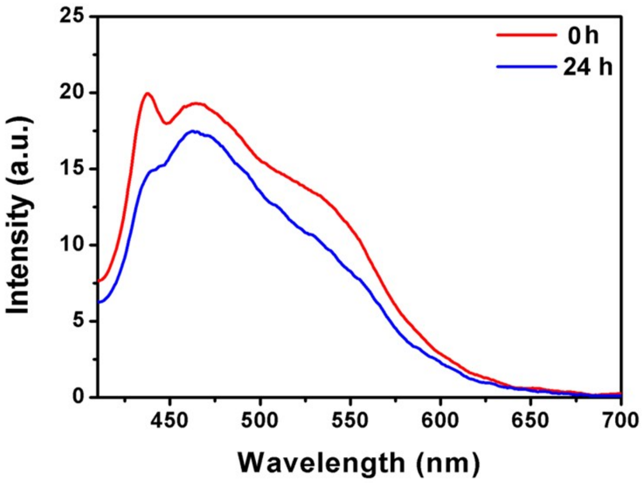

Figure S4. The fluorescence intensity of Ber-CDs in PBS (pH=5.5) for 0 and 24 h.

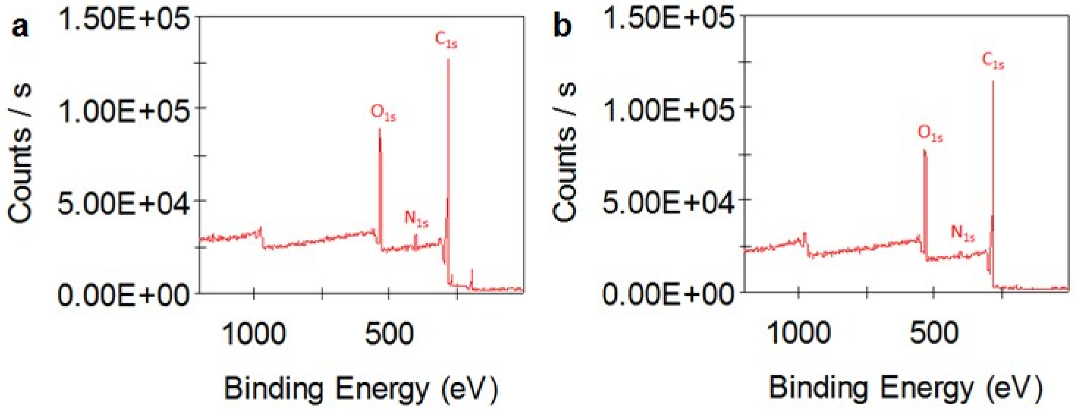

Figure S5. The XPS analyses of (a) berberine and (b) Ber-CDs.

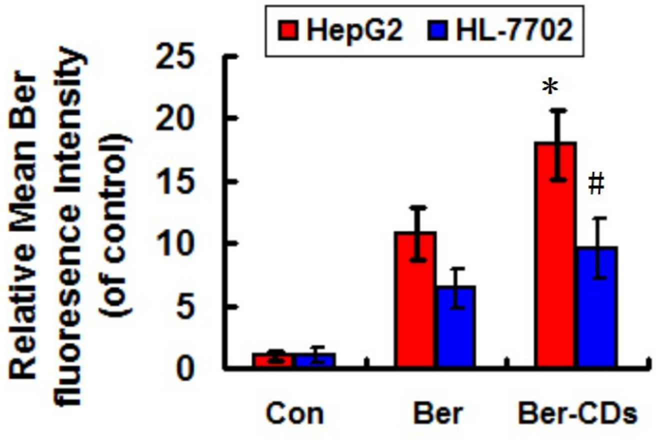

Figure S6. Quantitative analysis of the internalization of the Ber or Ber-CDs in the HepG2 cells and HL-7702 cells after 3 h of exposure using FACS. These data represent three separate experiments and are presented as the mean values ± SD, and $^{*}P < 0.05$ vs the Ber group, $^{\#}P < 0.05$ vs the HepG2 group.

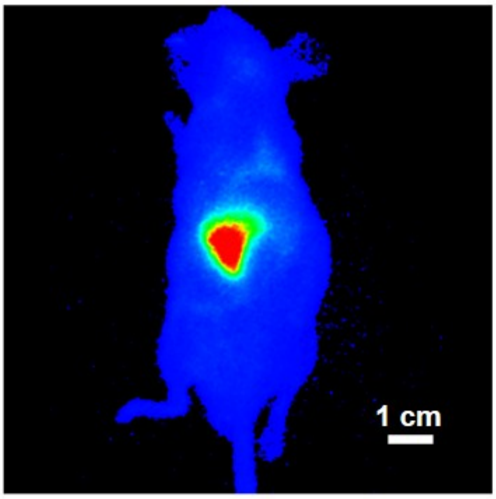

Figure S7. In vivo fluorescence bioimaging of Ber-CDs in HepG2 tumor-bearing mouse.

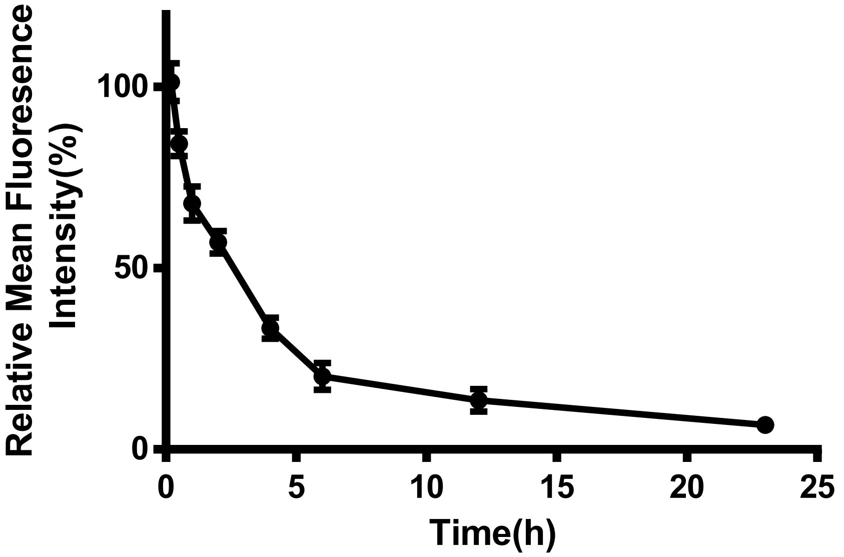

Figure S8. The blood circulation of Ber-CDs in vivo.

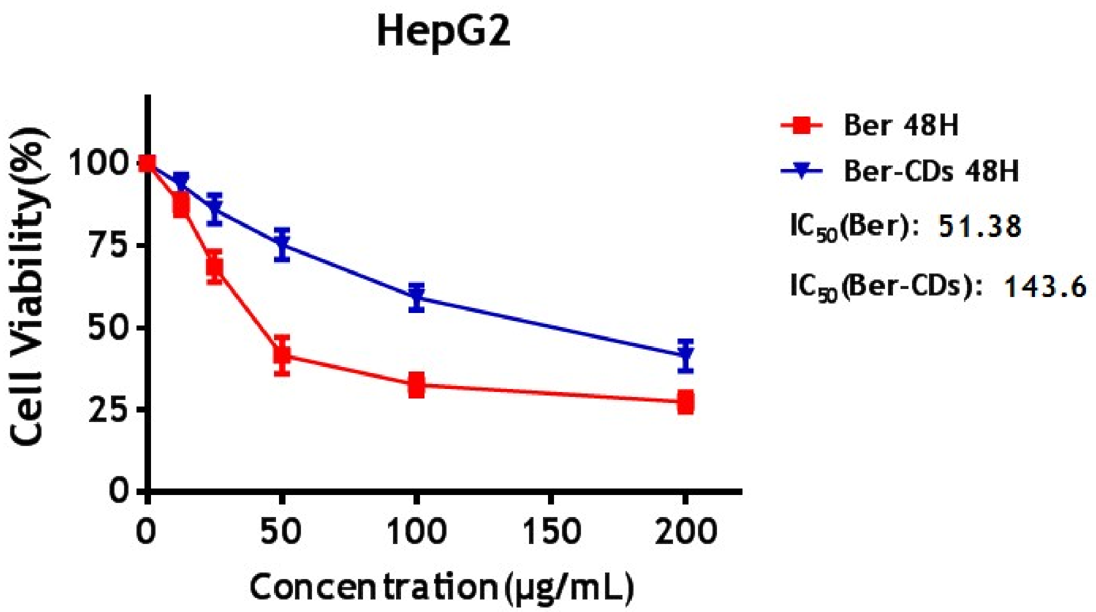

Figure S9. Cell viability of HepG2 cells incubated with various concentrations of Ber or Ber-CDs for 48 h. These data represent three separate experiments and are presented as the mean values ± SD.

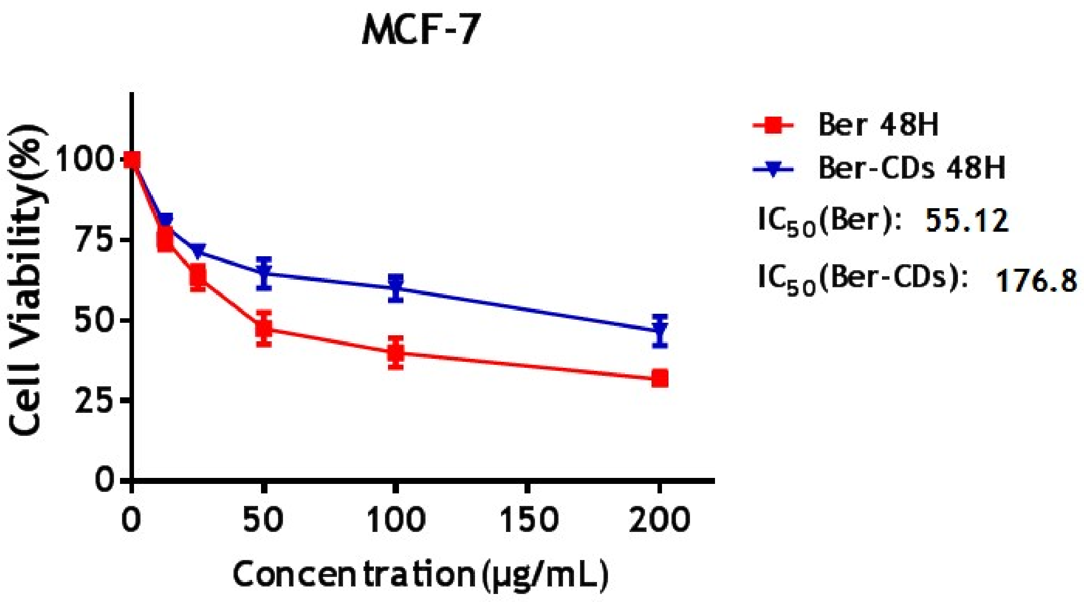

Figure S10. Cell viability of MCF-7 cells incubated with various concentrations of Ber or Ber-CDs for 48 h. These data represent three separate experiments and are presented as the mean values ± SD.

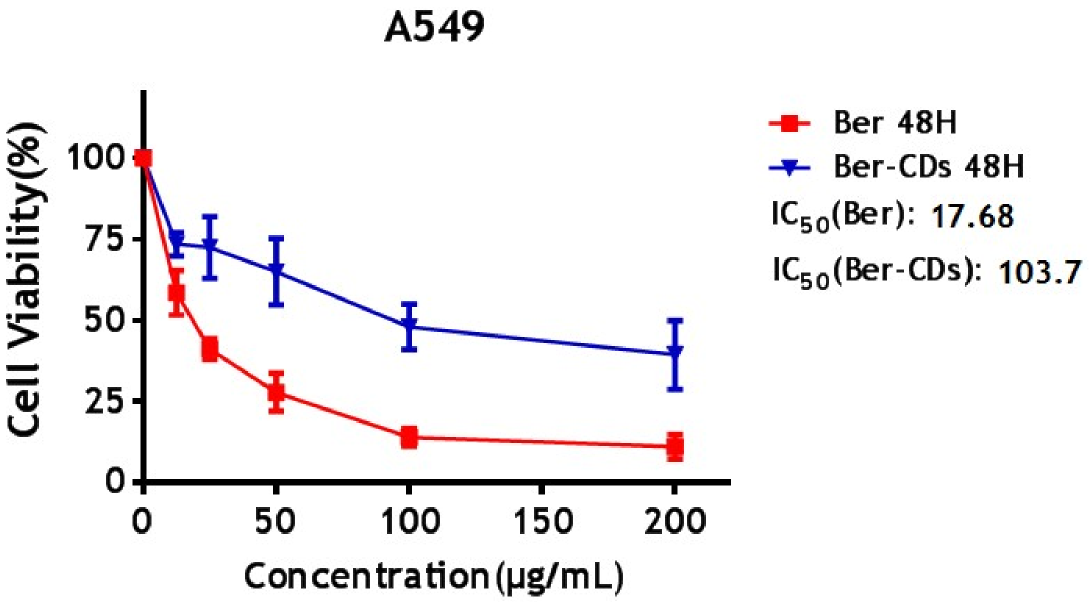

Figure S11. Cell viability of A549 cells incubated with various concentrations of Ber or Ber-CDs for 48 h. These data represent three separate experiments and are presented as the mean values ± SD.

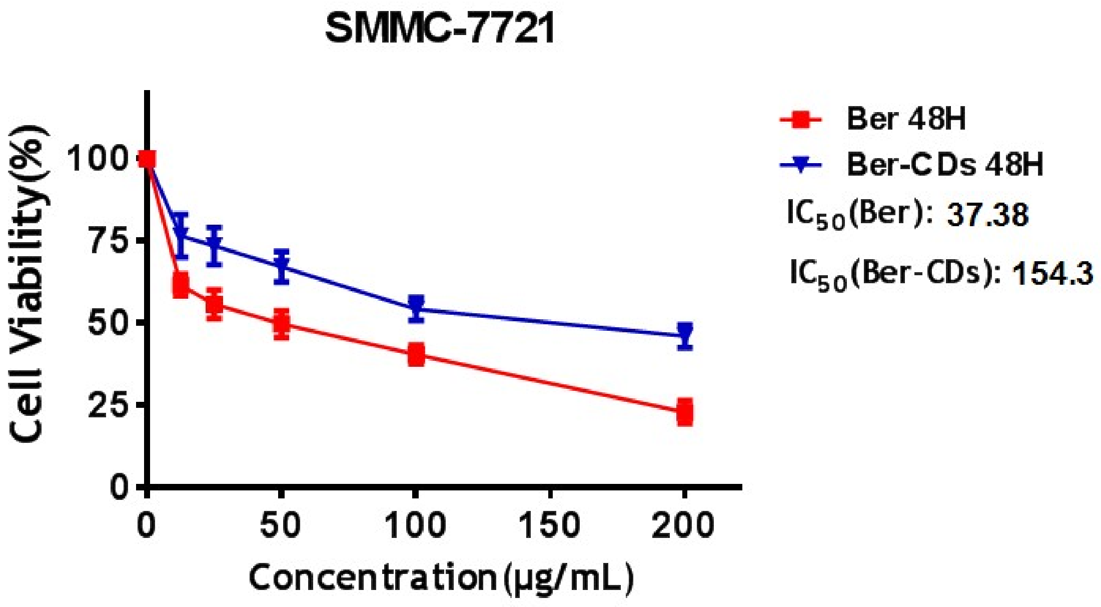

Figure S12. Cell viability of SMMC-7721 cells incubated with various concentrations of Ber or Ber-CDs for 48 h. These data represent three separate experiments and are presented as the mean values ± SD.

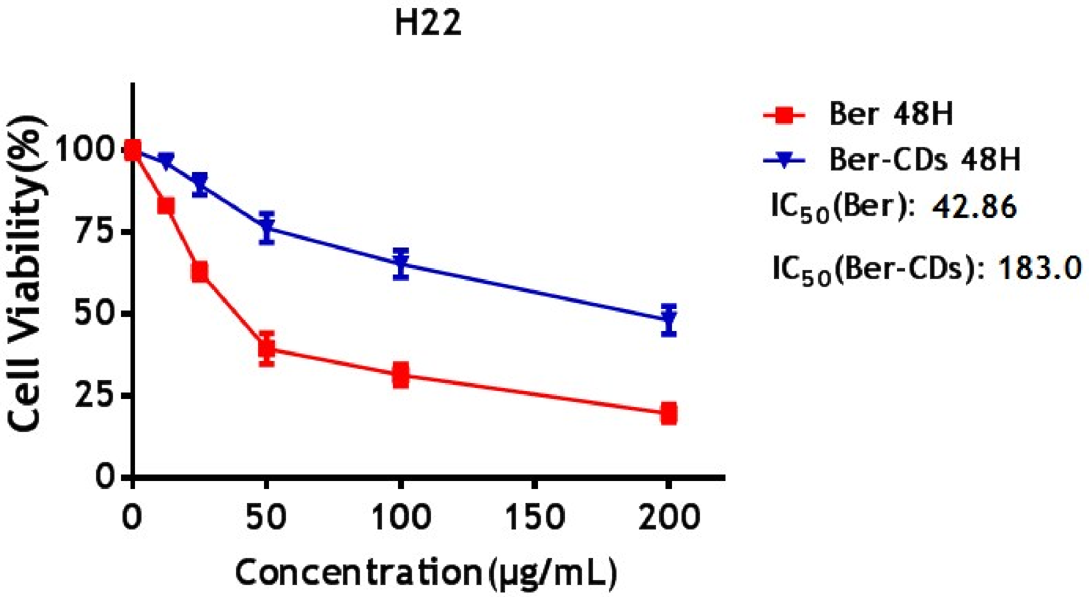

Figure S13. Cell viability of H22 cells incubated with various concentrations of Ber or Ber-CDs for 48 h. These data represent three separate experiments and are presented as the mean values ± SD.

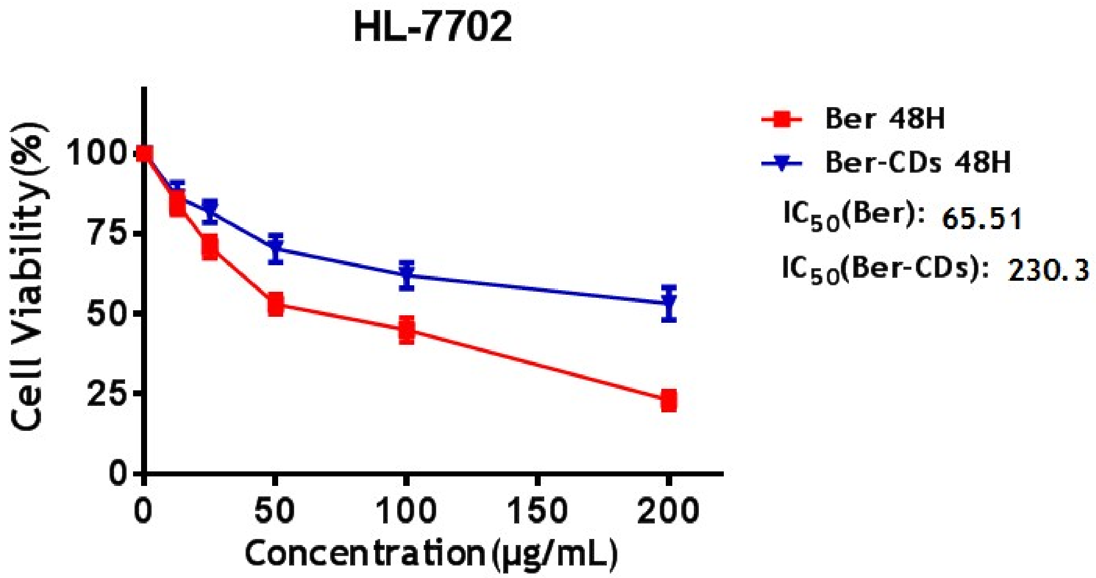

Figure S14. Cell viability of HL-7702 cells incubated with various concentrations of Ber or Ber-CDs for 48 h. These data represent three separate experiments and are presented as the mean values ± SD.

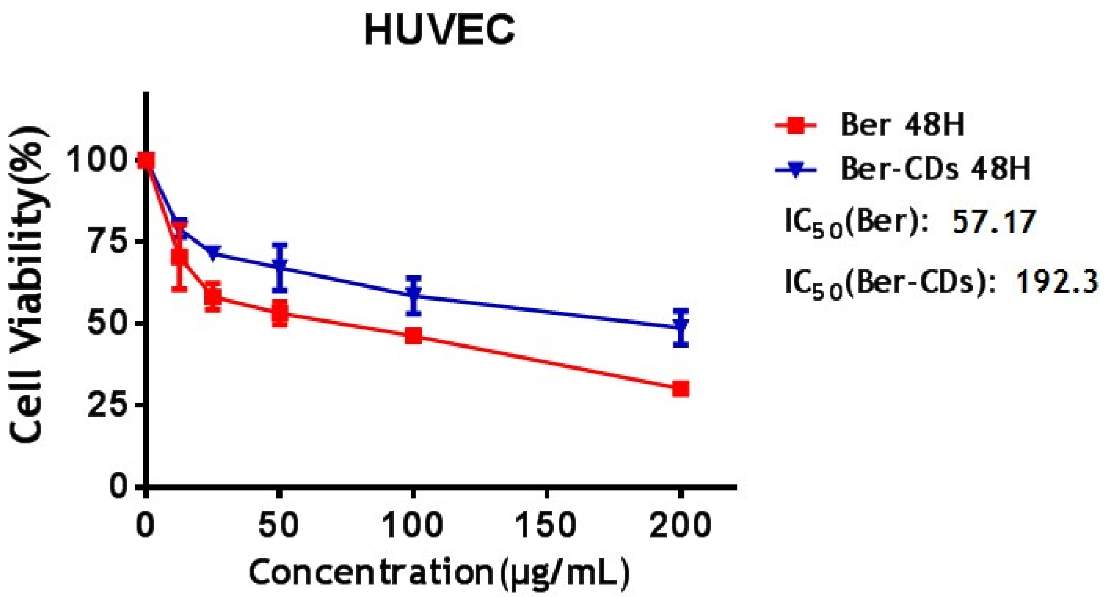

Figure S15. Cell viability of HUVEC cells incubated with various concentrations of Ber or Ber-CDs for 48 h. These data represent three separate experiments and are presented as the mean values ± SD.

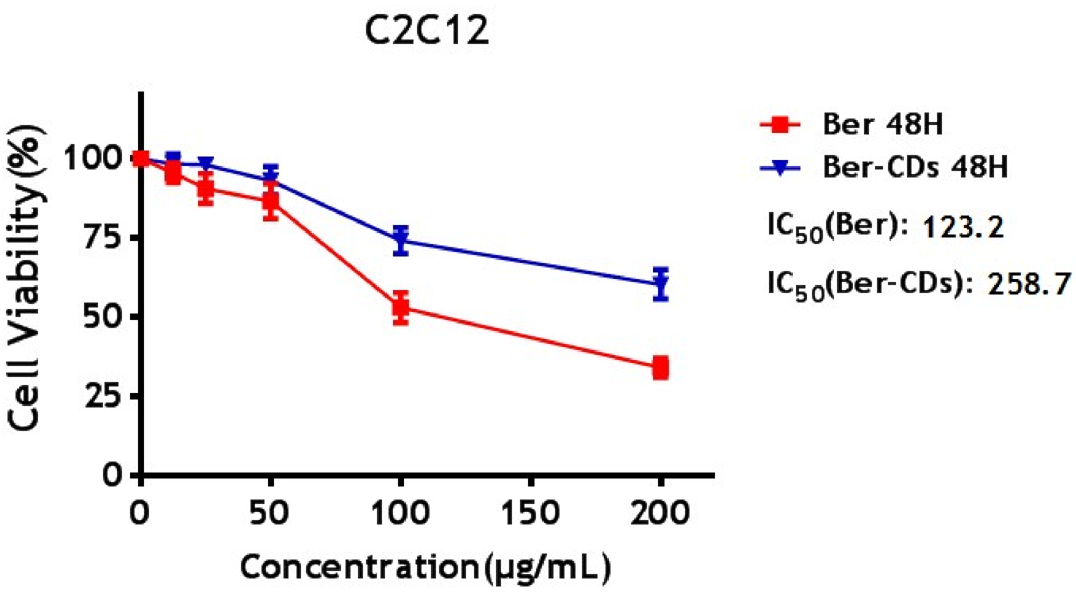

Figure S16. Cell viability of C212 cells incubated with various concentrations of Ber or Ber-CDs for 48 h. These data represent three separate experiments and are presented as the mean values ± SD.

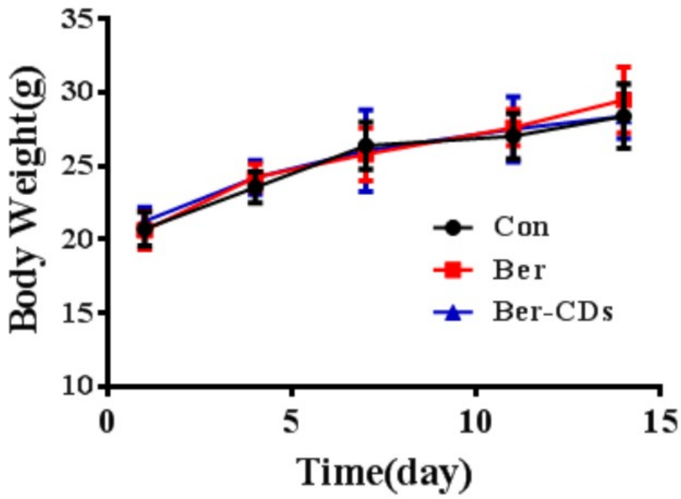

Figure S17. The body weights of H22 tumor-bearing mice in the control, Ber and Ber-CDs groups at 14 days. Each bar represents the mean ± SD, n=6.

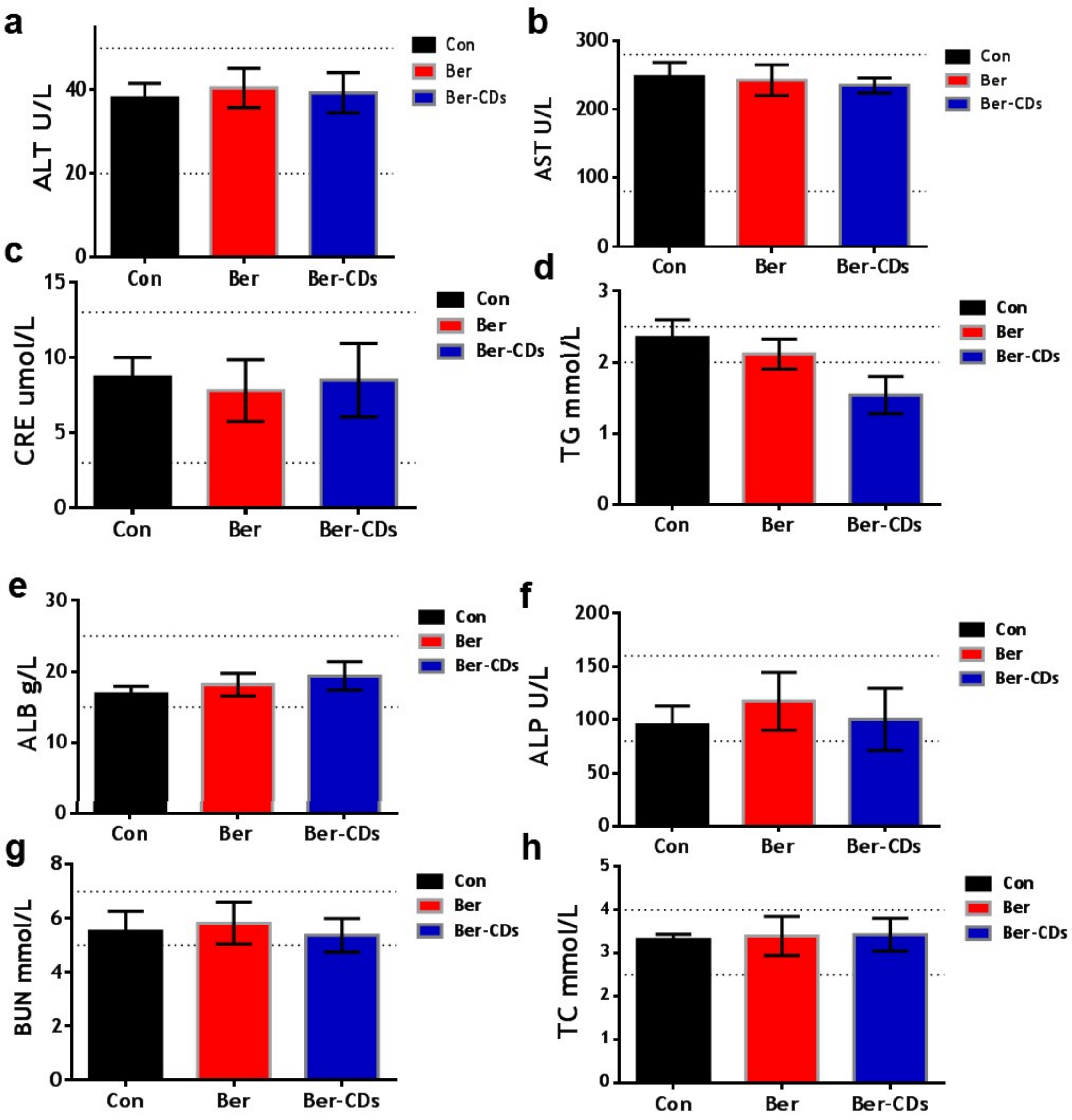

Figure S18. Biochemical parameters of mice in the control, Ber and Ber-CDs groups at 14 days. (a) alkaline aminotransferase (ALT), (b) aspartate phosphatase (AST), (c) creatinine (CRE), (d) triglyceride (TG), (e) albumin (ALB), (f) alkaline phosphatas (ALP), (g) blood urea nitrogen (BUN), and (h) total Cholesterol (TC). Each bar represents the mean ± SD, n=6.
